# *catena*-Poly[[dipyridine­nickel(II)]-*trans*-di-μ-chlorido] from powder data

**DOI:** 10.1107/S1600536810001820

**Published:** 2010-01-30

**Authors:** Edith Alig, Thomas Bernert, Lothar Fink, Nevzat Külcü, Tuncay Yeşilkaynak

**Affiliations:** a University of Frankfurt, Institute of Inorganic and Analytical Chemistry, Max-von-Laue-Strasse 7, 60438 Frankfurt am Main, Germany; b Mersin Universitesi, Ciftlikkoy Kampusu Fen-Edebiyat Fakultesi Kimya Bolumu, Mersin, Turkey

## Abstract

The asymetric unit of the title compound, [NiCl_2_(C_5_H_5_N)_2_]_*n*_, contains two Ni^II^ ions located on different twofold rotational axes, two chloride anions and two pyridine rings in general positions. Each Ni^II^ ion is coordinated by two pyridine rings, which form dihedral angles of 33.0 (2) and 11.0 (2)° for the two centers, and four chloride anions in a distorted octa­hedral geometry. The chloride anions bridge Ni^II^ ions related by translation along the short *b* axes into two crystallographically independent polymeric chains.

## Related literature

For the preparation of related compounds, see: Liptay *et al.* (1986 ▶). For related polymeric chains of octa­hedrally coordinated transition metal ions, see: Hu *et al.* (2003 ▶) and McConnell & Nuttall (1978 ▶). For the isostructural compound [CoCl_2_(C_5_H_5_N)_2_] with a detailed discussion of the pseudo-ortho­rhom­bic symmetry, see: Dunitz (1957 ▶). For details of the indexing algorithm, see: Boultif & Louër (1991 ▶). For details of Rietveld refinement, see: Young (1993 ▶).
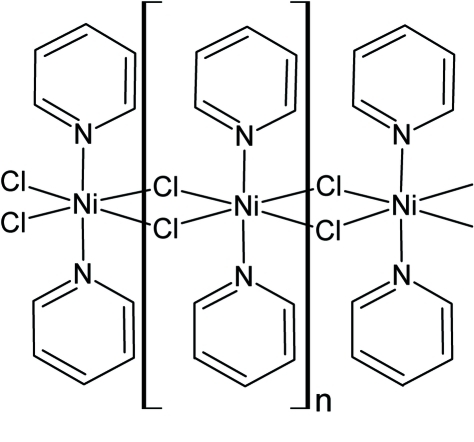


## Experimental

### 

#### Crystal data

[NiCl_2_(C_5_H_5_N)_2_]
*M* *_r_* = 287.79
Monoclinic, 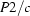

*a* = 19.2483 (4) Å
*b* = 3.62535 (4) Å
*c* = 17.3504 (2) Å
β = 116.883 (2)°
*V* = 1079.91 (3) Å^3^
*Z* = 4
Cu *K*α_1_ radiation
λ = 1.54056 Å
μ = 6.85 mm^−1^
*T* = 298 K
Cylinder, 12 × 0.5 mm

#### Data collection

Stoe Stadi-P diffractometer
Specimen mounting: specimen was sealed in a 0.5 mm diameter borosilicate glass capillary
Data collection mode: transmission
Scan method: step
2θ_min_ = 2°, 2θ_max_ = 110°, 2θ_step_ = 0.01°

#### Refinement

*R*_p_ = 0.024
*R*_wp_ = 0.032
*R*_exp_ = 0.028
*R*_Bragg_ = 0.009
χ^2^ = 1.357
10599 data points
126 parameters
61 restraints
H-atom parameters constrained

### 

Data collection: *WinXPOW* (Stoe & Cie, 2004 ▶); cell refinement: *DASH* (David **et al.**, 2004); data reduction: *WinXPOW* (Stoe & Cie, 2004 ▶); program(s) used to solve structure: *DASH*; program(s) used to refine structure: *TOPAS* (Coelho, 2007 ▶); molecular graphics: *Mercury* (Macrae *et al.*, 2006 ▶); software used to prepare material for publication: *PLATON* (Spek, 2009 ▶).

## Supplementary Material

Crystal structure: contains datablocks global, I. DOI: 10.1107/S1600536810001820/cv2682sup1.cif

Rietveld powder data: contains datablocks I. DOI: 10.1107/S1600536810001820/cv2682Isup2.rtv

Additional supplementary materials:  crystallographic information; 3D view; checkCIF report

## Figures and Tables

**Fig. 1.**
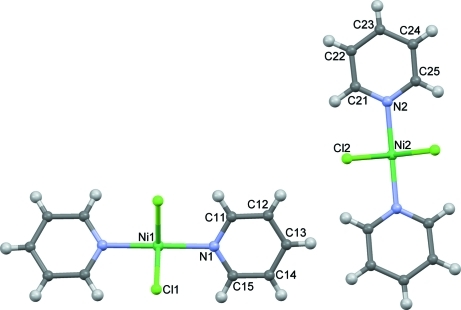
A portion of the crystal structure of (I) showing the atomic numbering of independent atoms and 50% probability displacement spheres.

**Fig. 2.**
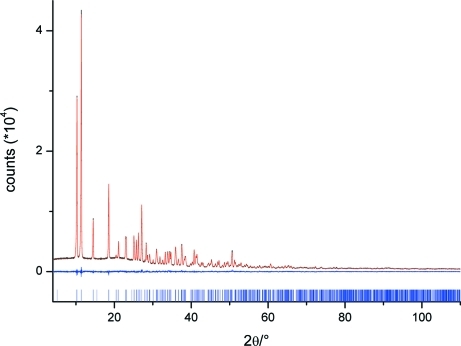
Experimental (black) and calculated (red) powder profiles of (I) with difference plot (blue).

## supplementary crystallographic information

### Comment

The title compound (I) was prepared by thermal decomposition of [NiCl_2_(C_5_H_5_N)_4_]. The product, [NiCl_2_(C_5_H_5_N)_2_], is isotypic with *trans*-[CoCl_2_(C_5_H_5_N)_2_] (Dunitz, 1957). The space group of the title compound was determined to *P*2/*c* with *a *= 19.24 Å, *b*= 3.63 Å, *c *= 17.35 Å, *β*= 116.82 ° and *Z* = 4. The four nickel atoms are located on two special positions (the twofold axes; Wyckoff positions 2*e* and 2*f*). Each nickel atom is coordinated by four chlorine atoms in the equatorial plane and two nitrogen atoms of the pyridine rings in axial positions. This leads to two different distorted coordination octahedra which are connected by edge sharing *via* bridging Cl atoms to build up two different one-dimensional chains. The distance between neighboured nickel atoms in each chain is equal to the lattice parameter *b*= 3.63 Å. An orthorhombic unit cell, found by DICVOL (Boultif & Louër, 1991), is related to the pseudo-orthorhombic cell for the isostructural compound *trans*-[CoCl_2_(C_5_H_5_N)_2_], which was discussed by Dunitz (1957). Similar as for the last compound, we found that the structure solution and refinement in orthorhombic symmetry does not lead to satisfying results.

### Experimental

[NiCl_2_(C_5_H_5_N)_4_] was heated to 400 K for 17 h (capillary, diameter: 0.5 mm).

### Refinement

Indexing with DICVOL (Boultif & Louër, 1991) led to two possible unit cells, a monoclinic and an orthorhombic one. The Pawley fit calculates nearly identical profile χ^2 ^values for both cells. The structure solution was carried out using simulated annealing with *DASH *(David *et al.*, 2004) and a modified molecular structure model based on [CoCl_2_(C_5_H_5_N)_2_] (Dunitz, 1957). The structure solution was tried in both crystal systems: monoclinic in *P*2/*c* with *a *= 19.24 Å, *b *= 3.63 Å, *c *= 17.35 Å, *β*= 116.82 ° and *Z *= 4 and several orthorhombic space groups with *C-*centered cells with *a *= 17.35 Å, *b *= 34.34 Å, *c *= 3.63 Å and *Z *= 8. As for [CoCl_2_(C_5_H_5_N)_2_] (Dunitz, 1957) the structure solution was succesful only for the monoclinic cell. The Rietveld refinement was carried out using TOPAS (Coelho, 2007) with Chebychev polynomial background correction and the pyridine rings restrained to be flat. Thermal parameters of non-hydrogen atoms were combined refined, except Ni. Thermal parameters of hydrogen atoms were constrained to those of the non-hydrogen atoms. The smooth difference curve (Fig. 2) shows that the structure is correct.

### Crystal data

**Table:**

[NiCl_2_(C_5_H_5_N)_2_]	*F*(000) = 584.0
*M**_r_* = 287.79	*D*_x_ = 1.770 Mg m^−^^3^
Monoclinic, *P*2/*c*	Cu *K*α_1_ radiation, λ = 1.54056 Å
Hall symbol: -P 2yc	µ = 6.85 mm^−^^1^
*a* = 19.2483 (4) Å	*T* = 298 K
*b* = 3.62535 (4) Å	Particle morphology: no specific habit
*c* = 17.3504 (2) Å	light green
β = 116.883 (2)°	cylinder, 12 × 0.5 mm
*V* = 1079.91 (3) Å^3^	Specimen preparation: Prepared at 400 K
*Z* = 4	

### Data collection

**Table:**

Stoe Stadi-P diffractometer	Data collection mode: transmission
Radiation source: X-ray tube	Scan method: step
primary focussing, Ge 111	2θ_min_ = 2°, 2θ_max_ = 110°, 2θ_step_ = 0.01°
Specimen mounting: Specimen was sealed in a 0.5 mm diameter borosilicate glass capillary	

### Refinement

**Table:**

Least-squares matrix: full with fixed elements per cycle	126 parameters
*R*_p_ = 0.024	61 restraints
*R*_wp_ = 0.032	3 constraints
*R*_exp_ = 0.028	H-atom parameters constrained
*R*_Bragg_ = 0.009	Weighting scheme based on measured s.u.'s *w* = 1/σ(Y_obs_)^2^
χ^2^ = 1.357	(Δ/σ)_max_ = 0.001
10599 data points	Background function: Chebychev polynomial
Excluded region(s): none	Preferred orientation correction: none
Profile function: modified Thompson–Cox–Hastings pseudo-Voigt (Young, 1993)	

### Fractional atomic coordinates and isotropic or equivalent isotropic displacement parameters (Å^2^)

**Table:**

	*x*	*y*	*z*	*U*_iso_*/*U*_eq_	
N1	0.08340 (13)	0.5786 (4)	0.19907 (14)	0.01963 (5)*	
Ni1	0	0.5639 (4)	0.25	0.02118 (10)*	
C11	0.15787 (11)	0.4942 (5)	0.25223 (11)	0.01963 (5)*	
C15	0.06141 (11)	0.6731 (5)	0.11471 (12)	0.01963 (5)*	
Cl1	−0.07443 (14)	1.0643 (6)	0.14865 (16)	0.01963 (5)*	
H11	0.1719 (7)	0.428 (3)	0.3113 (6)	0.02356 (6)*	
C12	0.21450 (11)	0.5012 (4)	0.22198 (12)	0.01963 (5)*	
C14	0.11707 (11)	0.6807 (5)	0.08359 (12)	0.01963 (5)*	
H15	0.0088 (6)	0.737 (4)	0.0767 (7)	0.02356 (6)*	
H12	0.2662 (6)	0.443 (3)	0.2583 (7)	0.02356 (6)*	
C13	0.19366 (11)	0.5934 (5)	0.13910 (12)	0.01963 (5)*	
H14	0.1030 (5)	0.740 (4)	0.0275 (7)	0.02356 (6)*	
H13	0.2309 (6)	0.599 (4)	0.1200 (7)	0.02356 (6)*	
N2	0.58174 (12)	0.1488 (4)	0.37857 (14)	0.01963 (5)*	
Ni2	0.5	0.1713 (5)	0.25	0.01780 (10)*	
C21	0.55807 (11)	0.1700 (5)	0.44079 (12)	0.01963 (5)*	
C25	0.65861 (11)	0.1073 (5)	0.39945 (12)	0.01963 (5)*	
Cl2	0.42724 (14)	0.6650 (5)	0.27908 (17)	0.01963 (5)*	
H21	0.5032 (6)	0.202 (4)	0.4254 (6)	0.02356 (6)*	
C22	0.61220 (10)	0.1492 (5)	0.52776 (12)	0.01963 (5)*	
C24	0.71442 (11)	0.0859 (5)	0.48673 (12)	0.01963 (5)*	
H25	0.6734 (6)	0.098 (4)	0.3546 (7)	0.02356 (6)*	
H22	0.5945 (6)	0.162 (4)	0.5697 (7)	0.02356 (6)*	
C23	0.68985 (11)	0.1072 (5)	0.55017 (12)	0.01963 (5)*	
H24	0.7655 (6)	0.057 (4)	0.5017 (7)	0.02356 (6)*	
H23	0.7259 (6)	0.097 (4)	0.6068 (7)	0.02356 (6)*	

### Geometric parameters (Å, °)

**Table:**

Ni1—Cl1	2.481 (2)	C12—C13	1.349 (3)
Ni2—Cl2	2.461 (3)	C14—C13	1.385 (2)
N1—Ni1	2.155 (3)	C14—H14	0.91 (1)
N1—C11	1.343 (2)	C13—H13	0.92 (1)
N1—C15	1.371 (3)	C21—H21	0.97 (1)
N2—Ni2	2.070 (2)	C21—C22	1.395 (2)
N2—C21	1.350 (4)	C25—C24	1.408 (2)
N2—C25	1.363 (3)	C25—H25	0.94 (1)
C11—H11	0.96 (1)	C22—H22	0.93 (1)
C11—C12	1.408 (3)	C22—C23	1.372 (3)
C15—C14	1.401 (4)	C24—C23	1.383 (4)
C15—H15	0.954 (9)	C24—H24	0.90 (1)
C12—H12	0.93 (1)	C23—H23	0.912 (9)
			
Ni1—N1—C15	121.1 (2)	C11—C12—H12	121.0 (7)
Ni1—Cl1—Ni1	93.8 (1)	C11—C12—C13	119.7 (2)
Ni1—N1—C11	118.3 (2)	C11—N1—C15	120.6 (2)
Ni2—N2—C21	119.5 (2)	C12—C13—C14	120.3 (2)
Ni2—N2—C25	119.7 (2)	C12—C13—H13	119.2 (7)
Ni2—Cl2—Ni2	94.0 (1)	C13—C14—H14	120.4 (7)
N1—Ni1—Cl1	89.4 (1)	C14—C15—H15	119.1 (7)
N1—Ni1—N1	177.1 (1)	C14—C13—H13	120.3 (7)
N1—C11—C12	120.4 (2)	C15—C14—C13	119.0 (2)
N1—C15—C14	119.7 (2)	C15—C14—H14	120.5 (7)
N1—C15—H15	121.2 (7)	C21—N2—C25	120.8 (2)
N1—C11—H11	119.0 (7)	C21—C22—H22	118.9 (8)
N2—C21—H21	120.3 (7)	C21—C22—C23	119.84 (19)
N2—C21—C22	120.3 (2)	C22—C23—C24	120.1 (2)
N2—C25—C24	120.0 (2)	C22—C23—H23	120.7 (8)
N2—C25—H25	118.8 (8)	C23—C24—H24	120.6 (8)
N2—Ni2—Cl2	91.9 (1)	C23—C24—H24	120.6 (8)
N2—Ni2—N2	175.5 (1)	C24—C25—H25	120.5 (8)
Cl1—Ni1—Cl1	86.1 (1)	C24—C23—H23	119.2 (8)
Cl1—Ni1—Cl1	179.9 (1)	C25—C24—C23	118.9 (2)
Cl1—Ni1—Cl1	93.8 (1)	C25—C24—H24	120.8 (8)
Cl1—Ni1—Cl1	86.2 (1)	H11—C11—C12	119.7 (7)
Cl2—Ni2—Cl2	86.7 (1)	H12—C12—C13	119.3 (7)
Cl2—Ni2—Cl2	179.3 (1)	H22—C22—C23	121.3 (8)
Cl2—Ni2—Cl2	94.0 (1)	H21—C21—C22	119.1 (7)
Cl2—Ni2—Cl2	85.3 (1)		
